# Identifying Small-Molecule Inhibitors of SARS-CoV-2 RNA-Dependent RNA Polymerase by Establishing a Fluorometric Assay

**DOI:** 10.3389/fimmu.2022.844749

**Published:** 2022-04-07

**Authors:** Xiaoming Bai, Hongmin Sun, Shuo Wu, Yuhuan Li, Lifei Wang, Bin Hong

**Affiliations:** ^1^ National Health Commission of the People's Republic of China (NHC) Key Laboratory of Biotechnology of Antibiotics, Institute of Medicinal Biotechnology, Chinese Academy of Medical Sciences and Peking Union Medical College, Beijing, China; ^2^ Chinese Academy of Medical Sciences (CAMS) Key Laboratory of Synthetic Biology for Drug Innovation, Institute of Medicinal Biotechnology, Chinese Academy of Medical Sciences and Peking Union Medical College, Beijing, China; ^3^ Chinese Academy of Medical Sciences (CAMS) Key Laboratory of Antiviral Drug Research, Institute of Medicinal Biotechnology, Chinese Academy of Medical Sciences and Peking Union Medical College, Beijing, China

**Keywords:** SARS-CoV-2, RNA-dependent RNA polymerase (RdRp), non-nucleoside analog inhibitors (NNAIs), high-throughput screening (HTS), nucleoside analogs (NAs)

## Abstract

SARS-CoV-2 (severe acute respiratory syndrome coronavirus‐2), a member of the coronavirus family, appeared in 2019 and has caused the largest global public health and economic emergency in recent history, affecting almost all sectors of society. SARS-CoV-2 is a single-stranded positive-sense RNA virus that relies on RNA‐dependent RNA polymerase (RdRp) activity in viral transcription and replication. Due to its high sequence and structural conservation in coronavirus and new SARS-CoV-2 variants, RdRp has been recognized as the key therapeutic target to design novel antiviral strategies. Nucleotide analogs (NAs), such as remdesivir, is the most promising class of RdRp inhibitors to be used in the treatment of COVID-19. However, the presence of exonucleases in SARS-CoV-2 caused a great challenge to NAs; the excision of incorporated NAs will lead to viral resistance to this group of inhibitors. Here, we expressed active RdRp protein in both a eukaryotic expression system of baculovirus-infected insect cells and a prokaryotic expression system of *Escherichia coli* cells. Nsp7 and nsp8 of the functional RdRp holoenzyme were generated in *E. coli*. An *in vitro* RdRp activity assay has been established with a reconstituted nsp12/nsp7/nsp8 complex and biotin-labeled self-priming RNAs, and the activity of the RdRp complex was determined by detecting binding and extension of RNAs. Moreover, to meet the needs of high-throughput drug screening, we developed a fluorometric approach based on dsRNA quantification to assess the catalytic activity of the RdRp complex, which is also suitable for testing in 96-well plates. We demonstrated that the active triphosphate form of remdesivir (RTP) and several reported non-nucleotide analog viral polymerase inhibitors blocked the RdRp in the *in vitro* RdRp activity assay and high-throughput screening model. This high-throughput screening model has been applied to a custom synthetic chemical and natural product library of thousands of compounds for screening SARS-CoV-2 RdRp inhibitors. Our efficient RdRp inhibitor discovery system provides a powerful platform for the screening, validation, and evaluation of novel antiviral molecules targeting SARS-CoV-2 RdRp, particularly for non-nucleotide antivirals drugs (NNAs).

## Introduction

In the past two decades, there have been three major coronavirus infection events worldwide. SARS-CoV-2 (severe acute respiratory syndrome coronavirus‐2), as the most highly pathogenic human coronavirus compared to SARS-CoV and MERS-CoV (1), has caused the largest global public health and economic emergency in recent history. As of March 6, 2022, SARS-CoV-2 had already spread to more than 216 countries worldwide with 440,807,756 infections and 5,978,096 deaths (2). The clinical use of multiple anti-SARS-CoV-2 drugs has been urgently approved in various countries, such as remdesivir, favipiravir, lopinavir/ritonavir, and chloroquine. Unfortunately, despite growing studies, there is no effective drug or treatment for COVID-19, and some antiviral drugs that have entered clinical trials still need to be carefully evaluated (3). Meanwhile, several WHO-approved vaccines have displayed varying degrees of effectiveness against SARS-CoV-2 in clinical trials. However, the developed vaccines do not ensure effectiveness against increasing variants of SARS-CoV-2.

SARS-CoV-2 is a positive-sense single-strand RNA virus with a ~30-Kb genome, including 14 open reading frames (ORFs), encoding 29 proteins. Replication and transcription of viral genome depend on the replication–transcription complex composed of an RNA-dependent RNA polymerase (RdRp) complex, nsp13 helicase (4–6), and several RNA-processing enzymes like nsp14 (7). The SARS-CoV-2 RdRp complex, containing an nsp12 catalytic core unit and two accessory factors nsp7 and nsp8, was previously reported as the minimal core component for virus RNA replicative machinery (8, 9). RdRp has become a key drug target against CoVs due to its essential role for viral replication, high sequence and structural conservation among CoVs, and the lack of a counterpart in human cells. Remdesivir (RDV), a nucleotide analog inhibitor of RdRp, is the first FDA-approved antiviral drug for COVID-19 treatment, displaying the broad spectrum of antiviral effects against numerous RNA viruses, including MERS-CoV and SARS-CoV (10). Nucleoside analogs (NAs) commonly target viral replication and insert into RNA by the RdRp complex, resulting in fatal mutations of the genome or termination of RNA synthesis. Besides remdesivir, several other nucleoside analogs like molnupiravir (11, 12), AT-527 (13), ritonavir (14), ribavirin (15, 16), favipiravir (15), and sofosbuvir (17, 18) are currently under study in clinical trials. The initial clinical results showed that RDV was superior to placebo in shortening the recovery time from viral infection. However, later research results sponsored by WHO and the National Institute of Allergy and Infectious Diseases showed that RDV treatment has no obvious improvement in the survival and outcomes of therapies. Therefore, WHO has insisted and issued a conditional recommendation against the use of RDV as per a November 20, 2020 report (19, 20). In the replication–transcription complex, it was reported that bifunctional enzyme nsp14 harboring both 3′-to-5′ ExoN and N7-methyltransferase activities can remove misincorporated nucleotides or nucleotide analogs from the nascent RNA (21–26). The proofreading activity of nsp14 causes SARS-CoV-2 resistance to RDV and may limit the development of nucleotide analog drugs for COVID-19 treatment. Therefore, discovery of non-nucleoside antivirals drugs (NNAs) targeting viral polymerase with high clinical efficacy and applicability to a broad range of patient statuses is highly required.

Here, we describe the expression and purification of active SARS-CoV-2 RdRp holoenzyme (nsp12/nsp7/nsp8) in both eukaryotic expression system of baculovirus-infected insect Sf9 cells and in prokaryotic system of *Escherichia coli* cells. With this reconstituted RdRp holoenzyme, we established RdRp activity assay *in vitro* to detect the binding and extension activity of RdRp complex to RNA primers for discovery of novel RdRp inhibitors. Moreover, we developed a fluorometric approach based on dsRNA quantification to assess the catalytic activity of the RdRp complex suitable for high-throughput screening (HTS). To investigate the reproducibility and reliability of this assay for expanded application in HTS, a pilot screen was conducted and the inhibitory effect of various RdRp inhibitors including nucleotide analogs and non-nucleotide analogs was evaluated. The results suggested that the newly established RdRp activity assay is suitable for the rapid and accurate screening of specific SARS-CoV-2 RdRp inhibitors.

## Materials and Methods

### RdRp Complex Protein Expression and Purification

The gene sequence of the SARS-CoV-2 nsp12 was optimized for expression in different host cells and then cloned into a modified pFastBac baculovirus expression vector containing a 5’ ATG starting sequence followed by a His tag coding sequence (GenScript, Beijing, China). Recombinant bacmid was transfected in *Spodoptera frugiperda* (Sf9) cells. After being amplified twice in Sf9 cells, 10 ml of recombinant baculovirus was used to infect 1 L of Sf9 cells at a density of 2–3×10^6^ cells per ml in Sf-900 II SFM media (GenScript) and incubated at 27°C in a humidified chamber for 48–72 h. Cells were collected and suspended in binding buffer (50 mM NaH_2_PO_4_, 300 mM NaCl, 50 mM imidazole, pH 7.4) at 4°C, and then lysed by sonication on ice. Cell lysate was centrifuged for 30 min at 13,000 rpm, and the supernatant and the pellet were collected, respectively. The supernatant of cell lysate was incubated with a Ni-NTA column (L00250/L00250-C) for 2 h. Then, the Ni-NTA column was washed with binding buffer until a baseline absorbance was achieved and protein was eluted with elution buffer (50 mM NaH_2_PO_4_, 300 mM NaCl, and 20–500 mM imidazole, pH 7.4). Fractions were analyzed by SDS-PAGE and Western blot. The high-purity fractions were pooled followed by 0.22-μm filter sterilization and stored at −80°C. Nsp12 gene was also cloned into a pGEX-6P-1 plasmid and expressed in *E. coli* fused to an additional GST tag at the N-terminus. The GST-nsp12 was co-expressed with molecular chaperone pg-Tf2 in LBBS medium (tryptone 10.0 g/L, yeast extract 5.0 g/L, NaCl 5.0 g/L, D-sorbitol 185.9 g/L, and betaine, 0.309 g/L). Bacterial cultures grew at 37°C in LB medium containing 100 μg/ml ampicillin and 20 μg/ml chloromycetin overnight and then were seeded in LBBS medium growing to an OD_600_ of 0.4. Isopropyl β-D-1-thiogalactopyranoside (IPTG) (0.1 mM) and tetracycline (5 ng/ml) were added to induce protein expression at 16°C for 60 h. Cell pellets were collected and resuspended in GST binding buffer (phosphate-buffered saline containing 1 mM DTT), lysed by MP FastPrep-24 5G (MP Biomedicals). Lysate was cleared by centrifugation of 4,700 rpm for 30 min and filtered with a 0.45-μm membrane. After being bound to GSTrap™HP (GE Healthcare), protein was eluted with GST elution buffer (50 mM Tris-HCl, 10 mM reduced glutathione, and 1 mM DTT, pH 8.0). Eluent was desalted by PD-10 Desalting Columns (GE Healthcare) in protein storage solution (phosphate-buffered saline containing 10% glycerin). The purified protein was packaged in Ultra-clean workbench and then stored at −80°C.

Nsp7, nsp8, and nsp7-His_6_-nsp8 were expressed in *E. coli* (Ec). The full-length genes coding nsp7, nsp8, and nsp7-His_6_-nsp8 were synthesized (Sangon Biotech, Beijing, China) and cloned into pET-22b (+) or pET-21a (+) vector, respectively. For protein expression, the vectors were transformed into *E. coli* BL21 (DE3). Bacterial cultures were grown to an OD_600_ of 0.6 at 37°C, and then the expression of protein was induced with a final concentration of 0.1 mM of IPTG at 16°C for 20–22 h. Cells were suspended in binding buffer (50 mM NaH_2_PO_4_, 300 mM NaCl, and 50 mM imidazole, pH 7.4) and lysed by MP FastPrep-24 5G (MP Biomedicals). Lysate was cleared by centrifugation of 4,700 rpm for 30 min and filtered with a 0.45-μm membrane. The filtrate was incubated with HisTrap™HP (GE Healthcare). After washing with binding buffer, the protein was eluted with elution buffer (50 mM NaH_2_PO_4_, 300 mM NaCl, and 300 mM imidazole, pH 7.4). Eluent was desalted by PD-10 Desalting Columns (GE Healthcare) in protein storage solution and stored at −80°C.

### SDS-PAGE and Western Blotting

Cells were lysed by FastPrep-24 5G (MP Biomedicals) and centrifuged at 13,000 rpm for 15 min. Lysate was separated on 8% (nsp12) or 12% (nsp7, nsp8, and nsp7H8) sodium dodecyl sulfate-polyacrylamide gels and stained directly with Coomassie G-250 according to standard protocols. As for Western blot assay, lysates were separated on SDS-PAGE and then transferred to polyvinylidene fluoride (PVDF) membranes (GE Healthcare). The membranes were blocked with 5% skim milk TBST for 120 min at room temperature, rinsed with TBST, and stained with antibodies against His tag (Cat no. 2366S, Lot no. 15, Cell Signaling Technology, Inc., Danvers, MA, USA) or GST tag (Cat no. 2368T, Lot no. 15, Cell Signaling Technology, Inc., Danvers, MA, USA) at 4°C for 12 h. After three rinses with TBST, membranes were incubated with HRP-conjugated secondary antibodies (Cat no. ZB-2305, ZB-2301, Lot no. Origene, Beijing, China) for 120 min, and detected by Immobilon Western Chemiluminescent HRP Substrate (Millipore Corporation, Billerica, MA, USA) using the ChemiDoc™ Imaging System (Bio-Rad Laboratories).

### Preparation of Template-Primer RNA

S1^*^/S2 RNA primer/template consisted of two short RNA oligonucleotides. One short RNA oligonucleotide with a sequence of 5’-bio-GCUAUGUGAGAUUAAGAAU U-3’ was used as the primer strand and another RNA oligonucleotide with a sequence of 5’-UUUUUUUUUUAAUUC UUA AUCUCACAUAGC-3’ was used as template strand. To anneal the RNA duplex, two oligonucleotides were mixed at equal molar ratio in annealing buffer (10 mM Tris-HCl, pH 8.0, 25 mM NaCl, and 2.5 mM EDTA), denatured by heating to 94°C for 5 min and then slowly cooled to room temperature. A long self-priming RNA contains a 20-nucleotide (nt) self-complementary base pairing region and a 20-nt RNA template (Ux20) with a sequence of 5’-Biotin-UUUUUUUUUUUUUUUUUUUUUUUUUUUUUUAACAGGUUCUAGAACCUGUU-3’ was used as the RdRp elongation assay template (27). All template-primer RNAs were synthesized by Sangon Biotech (Beijing, China).

### Electrophoretic Mobility Shift Assays

To verify the binding activity of the RdRp complex, an electrophoretic mobility shift assay was performed. The RdRp complex was preincubated at 4°C overnight and then incubated with S1^*^/S2 RNA primer/template in EMSA binding buffer (Thermo Fisher Scientific, Waltham, MA, USA) for 30 min at 30°C. Samples were resolved on 6% native polyacrylamide gels running in 0.5×TBE buffer at 120 V for 45 min in a 4°C room and then transferred to nylon membrane (Amersham Biosciences) running in 0.5×TBE buffer at 15 V for 40 min. The biotin-labeled RNA was detected by LightShift™ EMSA Optimization& Control Kit (Thermo Fisher Scientific, Waltham, MA, USA) according to the manufacturer’s protocol and imaged by the ChemiDoc™ Imaging System (Bio-Rad Laboratories).

### RdRp Enzymatic Activity Assay and Its Inhibition by Inhibitors

The SARS-CoV-2 RdRp complex (0, 0.5, 1.0, 1.5, 2.0, and 3.0 μM) was incubated with 3.0 nM biotin labeled self-priming RNA, 2 U/μl RNase inhibitor, and 10 mM ATP in reaction buffer (20 mM Tris, pH 8.0, 1 mM DTT, 6 mM MgCl_2_, 10 mM KCl, and 0.01% Triton-X 100) for 60 min at 37°C. The reactions were stopped by 40 μl of quench buffer (94% formamide, 30 mM EDTA, prepared with DEPC-treated water). The reaction products were loaded into a 10% Urea-PAGE denatured gel, running at 120 V for 1.5 h. After electrophoretic transfer to nylon membrane (Amersham Biosciences), the labeled RNA was detected by LightShift™ EMSA Optimization & Control Kit (Thermo Fisher Scientific, Waltham, MA, USA) according to the manufacturer’s protocol. The inhibition assays of RdRp inhibitors are similar to the above for the RdRp enzymatic assays, except inhibitors were added to reactions for 30 min before the addition of 10 mM ATP.

### High-Throughput Fluorometric RdRp Assay With Self-Priming RNA

A long self-priming RNA with a sequence of 5’-Biotin-UUUUUUUUUUUUUUUUUUUUUUUUUUUUUUAACAGGUUCUAGAACCUGUU-3’ was used as the fluorometric RdRp assay template. The reaction system of RdRp enzymatic activity assay was modified for high-throughput assay. After adding compounds for 30 min, 2.0 μM SARS-CoV-2 RdRp was incubated for 60 min with 1.0 μM self-priming RNA and 10 mM ATP in the presence of 2 U/μl RNase inhibitor in reaction buffer (20 mM Tris, pH 8.0, 1 mM DTT, 6 mM MgCl_2_, 10 mM KCl, and 0.01% Triton-X 100). Formation of dsRNA is assessed by the QuantiFluor dsRNA system (Promega). This is followed by the addition of 200 μl of QuantiFluor dsRNA dye mix and incubation for 5 min at room temperature according to the manufacturer’s recommendation. Increased fluorescence was detected using 2030 Multilabel Reader (PerkinElmer) at an excitation of 504 nm and an emission of 531 nm.

### Calculation of *Z*-Factor and *Z’*-Factor

The concentration of total dsRNA was calculated using a linear regression model for each compound as the activity (*A*) of SARS-CoV-2 RdRp. *A* for each compound is normalized against the DMSO controls in each plate as follows:


Normalised_A=A/mean(A_DMSO)

where mean (*A*_DMSO) is the average of activity values of DMSO controls in a particular plate

To assess the quality of the screen, the *Z*-factor was calculated for C646 as follows.

To assess the reliability and reproducibility of the developed assay, *Z*-factor and *Z*’-factor values were evaluated using the method of Zhang et al. (28) First, we executed the assay as mentioned previously, and the experimental grouping was as follows: (1) negative group (*n* = 21 wells), without ATP, treated with 0.1% DMSO for 30 min; (2) positive group (*n* = 21 wells), treated with 0.1% DMSO for 30 min; and (3) inhibitor group (*n* = 21 wells), treated with 25 µM C646 for 30 min before adding ATP.

*Z*-factor was calculated using the following equation:


Z-Factor = 1  − [(3SDNegative + 3SDPositive)/|meanNegative− meanPositive|]

*Z*’-factor was calculated as follows:


Z’-Factor = 1  − [(3SDInhibitor + 3SDPositive)/|meanInhibitor− meanPositive|]

### Statistical Analysis

The data were presented as the mean ± SEM. All statistical data analyses were performed in GraphPad Prism 8. Statistical significance for each endpoint was determined with specific statistical tests. For each test, a *p*-value < 0.05 was considered significant. Specific tests to determine statistical significance are noted in each figure legend.

## Results

### Expression and Purification of SARS-CoV-2 RdRp Complex Proteins

**Figure 1:**
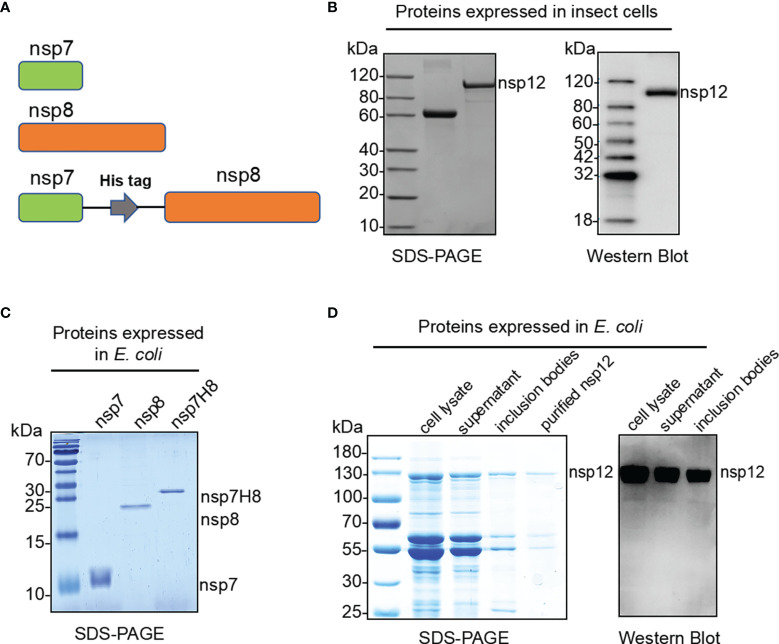
Expression and purification of the SARS-CoV-2 RdRp complex. **(A)** Schematic diagram for the components of the RdRp complex, containing nsp7, nsp8, and nsp7-His_6_-nsp8 (nsp7H8). **(B)** Purified SARS-CoV-2 nsp12 proteins expressed in baculovirus-infected insect cells (Sf9) were analyzed by SDS-PAGE and Western blot. **(C)** Bacterially expressed and purified SARS-CoV-2 nsp7, nsp8, and nsp7H8 proteins were analyzed by SDS-PAGE. **(D)** Bacterially expressed and purified SARS-CoV-2 nsp12 protein was analyzed by SDS-PAGE and Western blot.

The nsp12 protein is an essential component of the RdRp complex of the coronavirus replication and transcription (29). To perform its full function, nsp12 requires two accessory factors nsp7 and nsp8 (**Figure 1A**), which possibly enhance RdRp complex template binding and processivity. The SARS-CoV-2 RdRp complex, which consists of a nsp12 catalytic core unit, two copies of nsp8, and one copy of nsp7 (nsp12/nsp8x2/nsp7), was previously reported as the minimal core components for virus RNA replicative machinery (30). We tried to express nsp12 protein in a eukaryotic expression system of baculovirus-infected insect cells and a prokaryotic expression system of *E. coli* cells, respectively. In the eukaryotic expression system, nsp12 with C-terminus tagged with 6xHis was produced and purified using immobilized metal affinity chromatography to ∼90% purity (**Figure 1B**). To produce nsp12 protein more economically, recombinant nsp12 proteins carrying affinity GST-tag were expressed in *E. coli* and were purified by affinity chromatography (**Figure 1D**). Although most of the recombinant GST-nsp12 protein was expressed in inclusion bodies and found at the precipitated pellet of the cell lysate, a small portion remained as a soluble form in the supernatant. Co-expression of GST-nsp12 with a chaperone appeared to yield a slightly higher expression level of the soluble protein (data not shown). To form the core RdRp complex, nsp7 and nsp8 subunits C-terminally tagged with 6xHis were expressed and purified in *E. coli* individually (**Figure 1C**). It was reported that a combination of nsp7/nsp8 fusion protein and nsp12 is most efficient in terms of RNA primer conversion rate (9); thus, we also expressed and purified the protein that fused nsp7 and nsp8 with a His_6_ tag linker as an nsp7-His_6_-nsp8 (nsp7H8) for further experiments (**Figures 1A, C**).

### The Purified RdRp Complex Binds RNA Primer/Template *In Vitro*

**Figure 2:**
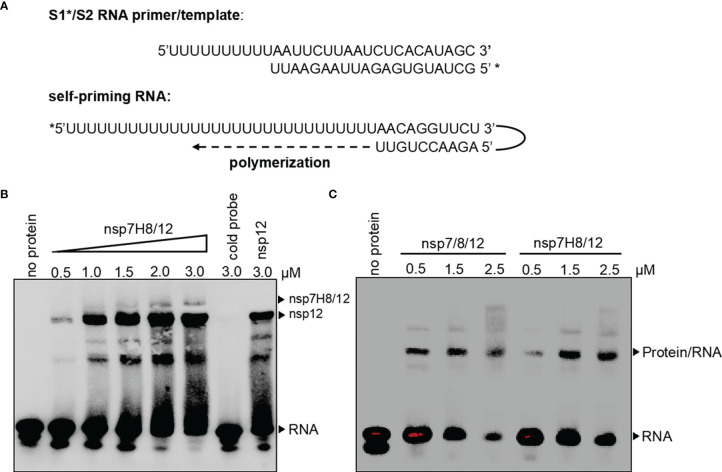
The RdRp complex binds with primer/template RNA. **(A)** Schematic diagram for a short S1^*^/S2 RNA primer/template and a long self-priming RNA. Primers which were labeled with biotin marked by *. **(B)** Gel mobility-shift assay of the interaction of the RdRp complex (nsp7H8/12) with S1^*^/S2 RNA primer/template. Lane “cold probe” contains 3 μM RdRp complex and 200-fold excess unlabeled competitor S1/S2 RNA. **(C)** Gel mobility-shift assays of the interaction of nsp7/8/12 or nsp7H8/12 with S1^*^/S2 RNA. Gel mobility-shift assays were performed using the different combinations of nsp7, nsp8, nsp7H8, and nsp12 protein purified from Sf9 cells. nsp7/8/12: His_6_-nsp7, His_6_-nsp8, and nsp12; 7H8/12: nsp7-His_6_-nsp8 and nsp12.

In order to catalyze RNA extension, core unit nsp12 needs to form an RdRp complex with subunits nsp7 and nsp8. To assess RdRp complex RNA binding and polymerization activity, we generated a short S1^*^/S2 RNA primer/template and a long self-priming RNA (**Figure 2A**). S1^*^/S2 is a primed RNA substrate by annealing the 20-nt 5′-biotin labeled (marked by *) primer S1 to the 30-nt template S2, while the long self-priming RNA contains a 20-nt self-complementary base pairing region and a 20-nt RNA template (Ux20). The short primer/template is more suitable for the binding test of RNA and protein (**Figure S1**), and the long self-priming primer is more suitable for distinguishing the extension of RNA. After incubation of the RdRp complex with primer/template RNA, reaction products were separated by native PAGE. We then compared the RNA binding properties of the nsp12, nsp7/8/12 (mol:mol:mol = 1:2:1), and nsp7H8/12 (mol:mol = 2:1) complex. The results showed that nsp12 itself can bind S1^*^/S2 RNA primer/template in electrophoretic mobility shift assays (EMSAs) (**Figure 2B**). Compared with nsp12 alone, when nsp7/8 or nsp7H8 was added to the EMSA reaction, a small portion of nsp12-RNA binding band shifted up, indicating that a larger RNA–protein complex formed, whereas nsp7/nsp8 maybe interacts weakly with RNA primer (**Figure 2B**). There were no binding bands with different strengths or sizes found between nsp7/8/12 and nsp7H8/12, suggesting that they have the same binding characteristics as S1^*^/S2 (**Figure 2C**). Our combined observations suggested that nsp7/8 and nsp7H8 appear to be inessential but may be helpful for the interaction of the polymerase with RNA.

### The RdRp Complex Catalyzes *In Vitro* RNA Synthesis

**Figure 3:**
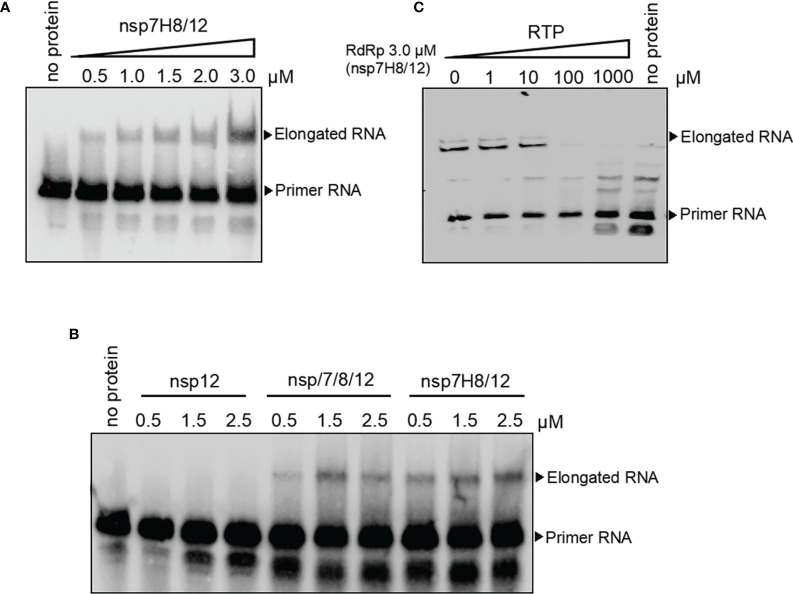
The RdRp complex catalyzes *in vitro* RNA synthesis. **(A)** Gel-based primer-extension assay to test RdRp complex (nsp7H8/12) catalytic activity. **(B)** Gel-based RdRp complex activity assay of nsp12, nsp7/8/12, and nsp7H8/12. **(C)** RdRp complex activity was effectively inhibited by the addition of the active triphosphate form RTP. RTP: triphosphate remdesivir. Primer extension polymerase assays were performed using the long self-priming RNA as primer/template RNA and different combinations of nsp7, nsp8, nsp7H8, and nsp12 protein purified from Sf9 cells. nsp7/8/12: His_6_-nsp7, His_6_-nsp8, and nsp12; nsp7H8/12: nsp7-His_6_-nsp8 and nsp12.

To assess RdRp complex activity of catalyzing RNA extension, we set up a polymerase extension reaction *in vitro*. RdRp preparation from insect cells containing C-terminally tagged nsp12 and nsp7 and nsp8 fusion protein (nsp7H8) (mol:mol = 1:2) was incubated at 4°C overnight to form the RdRp complex first. After incubation of the RdRp complex with self-priming RNA substrate and ATP, extension RNA products were separated by Urea-PAGE denatured gel. We found that the RdRp complex was able to extend the self-priming RNA substrate to generate duplex RNA in a dose-dependent manner, confirming RNA-dependent RNA polymerization activity (**Figure 3A**). We also noted that neither nsp12 nor nsp7H8 protein could extend the RNA primer alone (**Figures S2, S3**). The presence of nsp7 and nsp8 dramatically increased nsp12 catalytic activity to the primer/template RNA (**Figure 3B**). The nsp7H8/12 complex preparation from *E. coli* cells also readily catalyzed the RNA primer extension, whereas nsp12 alone did not (**Figure S2**), which is similar to the insect cell expressed nsp12 protein.

Remdesivir was known as an antiviral prodrug, which is one of the nucleotide analogs targeting virus replication. Remdesivir is converted to the active inhibitor for RdRp replication complex in the triphosphate form (RTP) within cells. We checked inhibition activity of RTP to RdRp catalytic activity *in vitro* subsequently. RdRp complex activity of *in vitro* RNA synthesis was effectively inhibited by the addition of the active triphosphate form, RTP (**Figure 3C**).

### Development of a Fluorometric RdRp Assay With Self-Priming RNA

**Figure 4:**
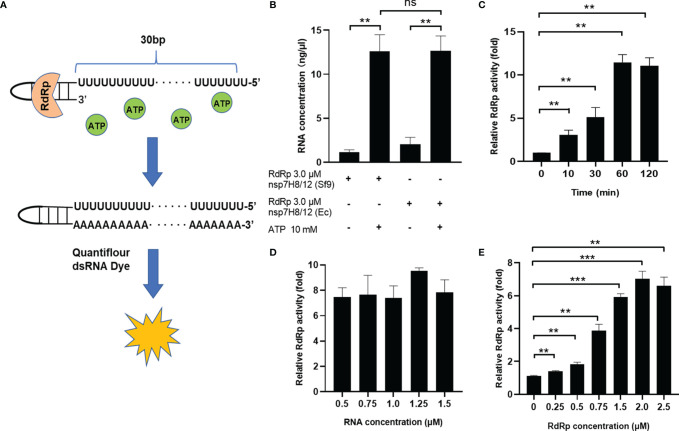
Development of a fluorometric RdRp assay with self-priming RNA. **(A)** Schematic diagram illustrating the fluorometric RdRp assay with self-priming RNA. The self-priming RNA-labeled 5’-biotin contains a 20-nt self-complementary base pairing region and a 20-nt RNA template (Ux20). RdRp activity detection assay is based on quantification of dsRNA by a fluorometric approach. The reaction system of RdRp catalytic primer extension was set up in a 96-well plate. **(B)** Activity of different RdRp preparations in the fluorometric RdRp activity assay. RdRp activity assay with optimized conditions including extension time **(C)**, RNA primer concentration **(D)**, and RdRp complex concentration **(E)**. The concentrations of dsRNA are assessed by the QuantiFluor dsRNA system (Promega) and normalized by dsRNA concentrations of negative control (without ATP). Data are presented as the mean ± SEM. Statistical significance was analyzed using one-tailed Student’s *t*-test. **p < 0.01; ***p < 0.001; ns, no significance. The data are representative of at least three independent experiments.

In order to meet the needs of HTS, we developed an efficient and reliable RdRp activity detection assay *in vitro* based on quantification of dsRNA by a fluorometric approach (**Figure 4A**). The reaction system of RdRp catalytic primer extension was set up in a 96-well plate with self-priming RNA-labeled 5’-biotin. It was reported that the QuantiFluor RNA system (Promega) can spot dsRNA content as low as 4 ng/ml and demonstrated up to 2.5 dsRNA/ssRNA signal ratio (31). Thus, in our assay, formation of dsRNA is assessed by the QuantiFluor dsRNA system. On the other hand, the QuantiFluor RNA system showed preference toward sense RNA, which is rich in uracil (U), we chose U-rich self-priming RNA for measurement of dsRNA formation from ssRNA following RdRp reaction (**Figure 4A**).

We tested the primer extension activity of nsp12 expressed from Sf9 cells and *E. coli* cells using the fluorometric RdRp activity detection assay in a protein concentration of 3.0 μM. As we expected, individual nsp12 or nsp7H8 did not show measurable primer-extension activity, but pre-incubating the nsp12 and nsp7H8 at a ratio of 1:2 showed significant activity (**Figure S3**). Nsp12 purified from different expression systems have the same catalytic activity (**Figure 4B**). To make RdRp activity assay suitable for high-throughput 96-well plate analysis, we optimized reaction conditions including extension time, RNA primer, and RdRp complex concentration to improve the sensitivity of the assay. We first checked the effect of different catalysis time on RNA primer extension, and the reactions were stopped at 0, 10, 30, 60, and 120 min after incubation with ATP. Our data showed that the RdRp complex was able to extend the primed RNA in a time-dependent manner until reaching its maximum activity at 60 min (**Figure 4C**). We also assessed the RNA primer concentration and observed the highest RdRp activity at 1.25 μM (**Figure 4D**). We further examined different ratios of the RdRp complex in a protein concentration of 0.25–2.5 μM at the above optimal conditions, and found that the optimum concentration of the RdRp complex in this system is 2.0 μM (**Figure 4E**). Together, a final concentration of 1.25 μM RNA primer and 2.0 μM RdRp complex in a 2:1 ratio of nsp7H8/12 and 60 min extension time was chosen and used for the high-throughput screen and all subsequent experiments. Finally, the presence of 5% DMSO, which is contributed by the chemical library in the HTS assay, did not affect RdRp activity (**Figure S4A**).

### Effects of Nucleoside Analogs and Non-Nucleoside Inhibitors on Fluorometric RdRp Assay

**Figure 5:**
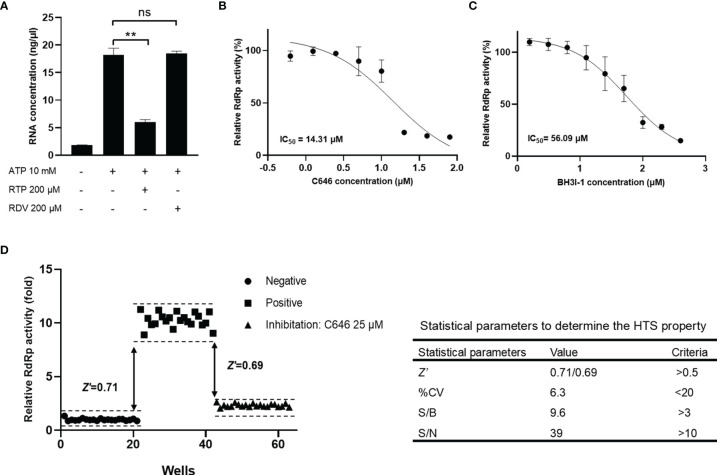
Reliability and reproducibility of the fluorometric SARS-CoV-2 RdRp activity assay system in HTS. The inhibition of remdesivir, triphosphate remdesivir **(A)**, C646 **(B)**, and BH3I1 **(C)** on SARS-CoV-2 RdRp activity in fluorometric RdRp activity assay. The data were presented as the mean ± SEM. Statistical significance was analyzed using one-tailed Student’s *t*-test and non-linear regression analysis of IC_50_ was conducted using GraphPad Prism^®^ Software V.8.0 for Windows (GraphPad Software Inc., San Diego, CA, USA). **p < 0.01; ns, no significance. The data are representative of at least three independent experiments. **(D)** Validation of the accuracy of the fluorometric SARS-CoV-2 RdRp activity assay as a high-throughput screening system. *Z*-factor was calculated using the relative RdRp activity obtained from the negative and positive groups to evaluate the discriminant ability of the assay for RdRp activity. *Z*’-factor was calculated using data obtained from the positive inhibitor group to evaluate the applicability of C646 as a positive control for the HTS assay.

To identify the utility of this optimized assay system in studying RdRp inhibitors, we checked well-known nucleoside analogs and non-nucleoside inhibitors targeting viral RNA polymerase, including remdesivir, ribavirin, molnupiravir, favipiravir, tenofovir, oxolinic acid, dasabuvir, C646, and BH3I1 (**Figure S7**). It is known that all antiviral nucleoside analogs targeting RdRp need to be metabolized into the 5′-triphostrates after entering the host cell, then compete with endogenous nucleotide triphosphates as substrates for viral RdRp. In a cell-free RdRp activity assay, NA inhibitors have to be modified into its active 5′-triphosphate form. Our data showed that compared with remdesivir (RDV), remdesivir triphosphate (RTP) can efficiently inhibit the SARS-CoV-2 RdRp in our assay (**Figure 5A**). C646 and BH3I1 were identified in recent studies as *in vitro* inhibitors of SARS-CoV-2 RdRp with no typical chemical structures of nucleoside analogs. As expected, both C646 and BH3I1 showed significant inhibition to SARS-CoV-2 RdRp activity with an IC_50_ value of 14.31 μM and 56.09 μM (**Figures 5B, C**). Despite acting as an active non-nucleoside analog inhibitor of MERS-CoV RdRp and HCV NS5B (32), dasabuvir only had weak inhibitory effects on SARS-CoV RdRp activity in our assay (**Figure S5A**). Not surprisingly, other NA inhibitors did not significantly inhibit SARS-CoV-2 RdRp activity in our assay because they were not in the form of triphosphate (**Figures S5B, C**).

### Reliability and Reproducibility of the Fluorometric SARS-CoV-2 RdRp Activity Assay System in HTS

Given that the activity of RdRp produced in *E. coli* (GST-tagged nsp12) and in insect cells (His-tagged nsp12) is closely similar (**Figures 4B**, **S2**), and GST protein alone had no RNA catalytic activity (**Figure S4B**), we used the *E. coli* expressed GST-tagged nsp12 to screen inhibitors of SARS-CoV-2 RdRp in the HTS assay. The *Z*- and *Z’*-factor are widely used statistical parameters in the evaluation and validation of HTS experiments (33). In our study, *Z*-factor was calculated using the relative Luc activity obtained from the negative and positive groups to evaluate the discriminant ability of the assay for RdRp activity. In addition, *Z*’-factor was calculated using data obtained from the positive inhibitor group to evaluate the applicability of C646 as a positive control for the HTS assay. The obtained *Z*-factor and *Z*’-factor values (0.711 and 0.685, respectively), % CV, S/B, and S/N met the criteria required for HTS assays (**Figure 5D**). It indicated that the fluorometric SARS-CoV-2 RdRp activity assay system is reliable and reproducible to identify RdRp inhibitors. This HTS model has been applied to a custom synthetic chemical and natural product library of thousands of compounds. The normalized inhibition of about 700 compounds plotted against the compound number is shown in **Figure S6**.

## Discussion

The nsp12 subunit is the crucial component of RdRp of the coronavirus replicative machinery. Purified recombinant nsp12 is able to extend a homopolymeric primer-template substrate by a few dozen nucleotides *in vitro*. In order to obtain the maximum catalytic activity of nsp12, most of the expression of recombinant nsp12 of coronaviruses is carried out in a eukaryotic expression system of baculovirus-infected insect cells. There are also some reports about the successful expression and purification of RdRp in *E. coli*. A recent study by Diffley (34) showed that the catalytic activity of the SARS-CoV-2 complex expressed by the *E. coli* system was even higher than that of the eukaryotic system despite lower yield. Our work was initiated by successfully expressing the active SARS-CoV-2 nsp12 protein in eukaryotic insect Sf9 cells. To more economically produce nsp12 protein to meet the demand of HTS, we next tried to express the GST-tagged nsp12 protein in *E. coli*. Unfortunately, recombinant GST-nsp12 was mainly expressed as inclusion bodies, instead of soluble forms. Therefore, we co-expressed a molecular chaperone with GST-nsp12 to increase the soluble protein and then generated enough nsp12 from supernatant for subsequent HTS assay. The *E. coli* expressed GST-nsp12 protein has a similar activity of RNA primer binding and extension to the Sf9 expressed protein, and it also has good sensitivity and reproducibility in our HTS model (**Figure 4B**). To the best of our knowledge, this is the first study to successfully establish an *in vitro* SARS-CoV-2 RdRp activity HTS assay using the nsp12/nsp7/nsp8 proteins all produced by *E. coli*.

Coronavirus nsp8 subunit forms a hexadecameric complex with nsp7 acting as an RNA primase required for nsp12-mediated RNA synthesis (9, 35). Previous work reported that SARS-CoV nsp12 itself does not bind significantly to RNA, nsp7/8 interacts weakly with RNA, whereas nsp7/8/12 strongly binds to RNA (9). We also tested if nsp8 and nsp7 act as the co-factors of SARS-CoV-2 RdRp in RNA binding and RNA synthesis. In our protein–RNA binding EMSA experiment, both eukaryotic and prokaryotic nsp12 surprisingly bind RNA primer without nsp7/8. The reported crystal structure of the template-RTP RdRp complex showed that extensive protein–RNA interactions are observed between the template-primer RNA and nsp12, with a total of 29 residues from nsp12 directly participating in the binding of the RNA, while no RNA interactions are mediated by nsp7 or nsp8 (36). This is not only consistent with our study of coronavirus RdRp binding features, but also supports that our assay may reflect the relatively true RNA binding process driven by the SARS-CoV-2 RdRp complex. The discrepancy may originate from a different experimental approach for establishing a protein–RNA binding system. In our RNA extension experiment and HTS assay, SARS-CoV-2 RdRp cannot initiate RNA extension until adding viral nsp7 and nsp8. It is consistent with previous reports that nsp8 and nsp7 act as the co-factors of SARS-CoV-2 RdRp to promote RNA synthesis.

Coronaviruses harbor a unique RNA replication proofreading mechanism, which depends on the ExoN to decrease the mutation rate of the error-prone viral RdRp. This function is executed by a viral exoribonuclease (ExoN) nsp14/nsp10 complex. Nsp14 is a bifunctional protein with two distinct activities, an N-terminal 3’-to-5’ ExoN and a C-terminal N7-methyltransferase (N7-MTase), both critical for coronavirus life cycle. Nsp14 ExoN activity is activated and stabilized through the interaction with the nsp10 protein. The proofreading function of the nsp14/nsp10 complex has been shown to critically decrease CoV sensitivity to chain terminating and mutagenic nucleotides (12, 26, 37–39). This offers a possible explanation for the poor inhibition activity of NA inhibitors against SARS-CoV-2 that have been reported both *in vitro* and *in vivo*. Although remdesivir has become the first NA approved to be clinically used in the treatment of COVID-19, latest clinical trials suggested that it is not as effective as first thought (1, 40–42). Unlike NA inhibitors, NNAs are thought to act noncompetitively by binding to a hydrophobic pocket located near the polymerase catalytic site, resulting in inhibition of polymerase activity. Several NNAs have been studied in targeting viral polymerase against hepatitis C virus (HCV), ZIKA (43), and human immunodeficiency virus (HIV) infections. In recent studies, an NNA lycorine was reported to inhibit diverse coronavirus infections such as SARS-CoV, MERS-CoV, HCoV-NL63, HCoV-OC43 and SARS-CoV-2 both *in vitro* and *in vivo* (44–46). Therefore, it might be more promising and pressing to develop novel non-nucleoside analog drugs that bind to the SARS-CoV-2 RdRp complex and cause an allosteric inhibition of virus replication and transcription.

Using our HTS system, we tested nucleoside analogs such as ribavirin, molnupiravir, favipiravir, tenofovir, remdesivir, and oxolinic acid, as well as the non-nucleoside analogs dasabuvir, C646, and BH3I1. Among them, C646 and BH3I1 were identified in recent studies as *in vitro* inhibitors of SARS-CoV-2 RdRp with no typical chemical structures of nucleoside analogs. C646 is a competitive inhibitor of the histone acetyltransferase p300 (46–49) and also significantly suppresses the replication of different strains of influenza A viruses in A549 cells and murine models (50). BH3I-1 inhibits Bcl-xL heterodimerization *in vitro* and induces cytochrome C release (51). Both of these compounds significantly inhibit SARS-CoV-2 RdRp activity in our assay. Another non-nucleoside analog, dasabuvir, is a non-nucleoside inhibitor of the hepatitis C virus (HCV) non-structural protein 5B (NS5B). Despite also acting as an active inhibitor of MERS-CoV RdRp (52), dasabuvir only had weak inhibitory effects on SARS-CoV-2 RdRp activity in our assay. Meanwhile, in the case of nucleoside analogs such as ribavirin, molnupiravir, favipiravir, and tenofovir without 5’-triphosphate, they did not show inhibition of SARS-CoV-2 RdRp activity in our assay. Compared with remdesivir (RDV), remdesivir triphosphate (RTP) can efficiently inhibit the SARS-CoV-2 RdRp in our assay.

In summary, we have expressed and purified SARS-CoV-2 RdRp in two expression hosts, a eukaryotic system of baculovirus-infected insect Sf9 cells and a prokaryotic system of *E. coli* cells. RdRp co-factors nsp7 and nsp8 were also expressed using the *E. coli* system. Based on these purified proteins, we reconstituted the RNA–RdRp complex binding and extension reactions *in vitro* and optimized catalytic conditions to detect RdRp activity. For the application in HTS, a novel fluorometric SARS-CoV-2 RdRp activity assay, which combined a fluorometric-based dsRNA quantification assay with the optimized RNA–RdRp reaction, was developed to assess the catalytic activity of the RdRp complex in a 96-well plate. The detected activity of different kinds of RdRp inhibitors confirmed that our system is a reliable and useful HTS tool for screening specific and effective SARS-CoV-2 RdRp inhibitors. This screening strategy provides a valuable platform for the screening, validation, and evaluation of novel antiviral molecules targeting SARS-CoV-2 RdRp, particularly for non-nucleotide analogs.

## Data Availability Statement

The original contributions presented in the study are included in the article/**Supplementary Material**. Further inquiries can be directed to the corresponding authors.

## Author Contributions

XB and HS were responsible for data collection, statistical analysis, and article writing. SW and YL participated in statistical analysis. LW and BH designed the article and took part in writing and revising. All authors contributed to the article and approved the submitted version.

## Funding

This work was supported by grants from the National Natural Science Foundation of China (82104046 and 81630089), the Beijing Municipal Natural Science Foundation (7214286), and the CAMS Innovation Fund for Medical Sciences (2021-I2M-1-030 and 2020-I2M-2-010).

## Conflict of Interest

The authors declare that the research was conducted in the absence of any commercial or financial relationships that could be construed as a potential conflict of interest.

## Publisher’s Note

All claims expressed in this article are solely those of the authors and do not necessarily represent those of their affiliated organizations, or those of the publisher, the editors and the reviewers. Any product that may be evaluated in this article, or claim that may be made by its manufacturer, is not guaranteed or endorsed by the publisher.
