# Supplementary material for: Identifying Small-Molecule Inhibitors of SARS-CoV-2 RNA-Dependent RNA Polymerase by Establishing a Fluorometric Assay
Source: Front Immunol. 2022 Apr 7;13:844749. doi: 10.3389/fimmu.2022.844749 (PMC9021610; doi:10.3389/fimmu.2022.844749)
Supplement: Supplementary file 1 [file DataSheet_1.pdf]

Supplementary material
Supplementary Figures

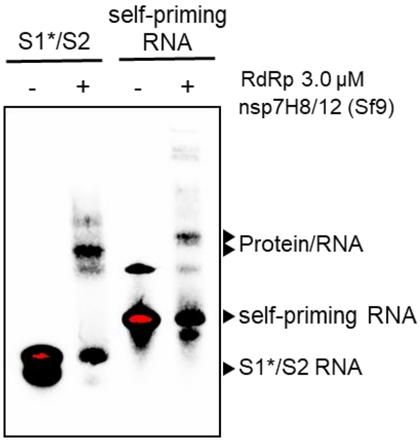

Figure S1. The RdRp complex purified from Sf9 cells binds with different primer/template RNA. nsp7H8/12: nsp7-His$_6$-nsp8 and nsp12.

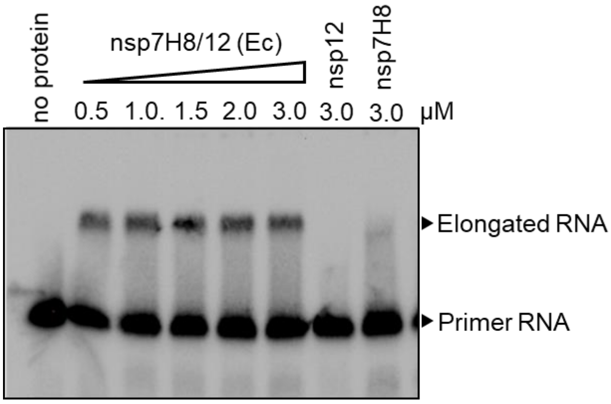

Figure S2. The RdRp complex purified from *E. coli* catalyzes *in vitro* RNA synthesis. nsp7H8: nsp7-His$_6$-nsp8; nsp7H8/12: nsp7-His$_6$-nsp8 and nsp12.

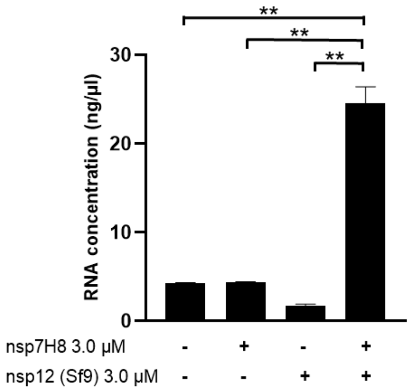

Figure S3. RNA extension activity of the individual nsp7H8 or nsp12 on the fluorometric RdRp activity assay. The data were presented as the mean ± SEM. Statistical significance was analyzed using one-tailed Student's *t*-test ** $p < 0.01$. The

data are representative of at least three independent experiments. nsp7H8: nsp7-His$_6$-nsp8.

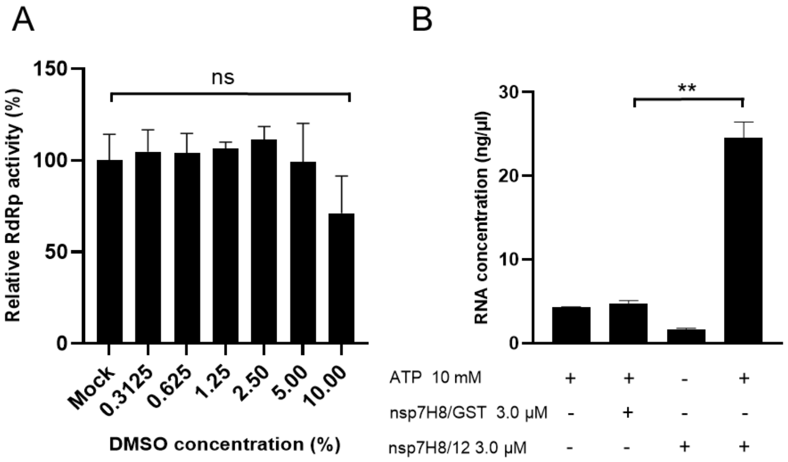

Figure S4. The effect of DMSO and GST protein on the fluorometric RdRp activity assay. The data were presented as the mean ± SEM. Statistical significance was analyzed using one-tailed Student's *t*-test. ** $p < 0.01$; ns: no significance. The data are representative of at least three independent experiments. nsp7H8/GST: nsp7-His$_6$-nsp8 and GST protein; nsp7H8/12: nsp7-His$_6$-nsp8 and nsp12.

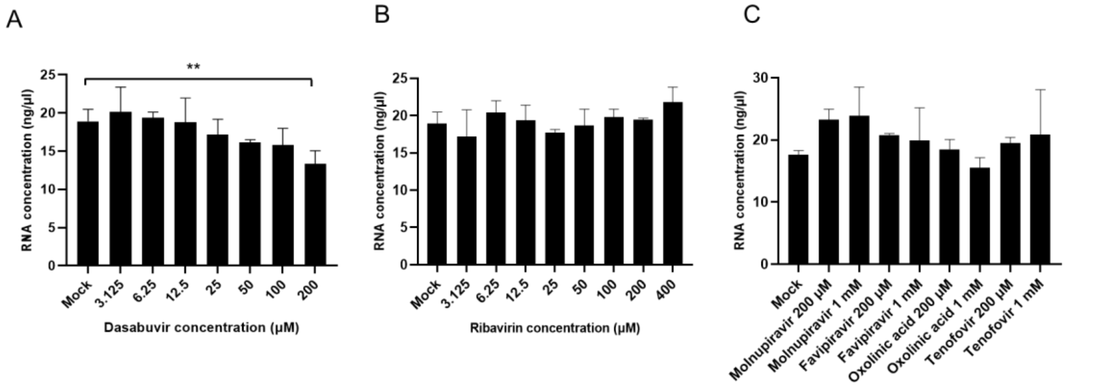

Figure S5. Effects of nucleoside analog and non-nucleoside analog inhibitors on SARS-COV-2 RdRp activity. The data were presented as the mean ± SEM. Statistical significance was analyzed using one-tailed Student's *t*-test. ** $p < 0.01$. The data are representative of at least three independent experiments.

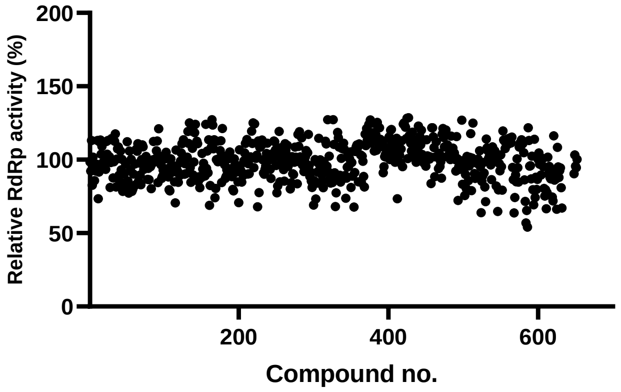

Figure S6. Results of the HTS screen of 700 compounds from a custom synthetic chemical and natural product library. HTS screen performed at compound concentrations of 25 μM.

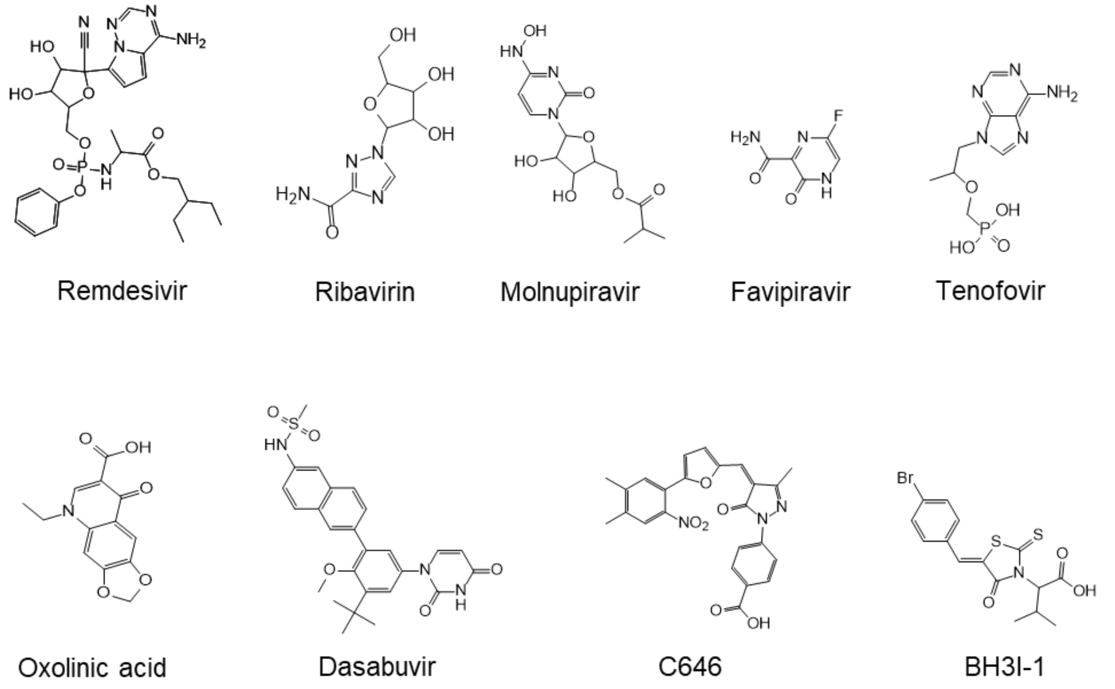

Figure S7. The chemical structures of some nucleoside analogs and non-nucleoside RdRp inhibitors.
